# Identification of two tandem genes associated with primary rosette branching in flowering Chinese cabbage

**DOI:** 10.3389/fpls.2022.1083528

**Published:** 2022-12-19

**Authors:** Jian Guan, Jinyan Li, Qingyu Yao, Zhiyong Liu, Hui Feng, Yun Zhang

**Affiliations:** College of Horticulture, Shenyang Agricultural University, Shenyang, China

**Keywords:** flowering Chinese cabbage, primary rosette branches, gene identification, tandem genes, GA2ox1 oxidase

## Abstract

Branching is an important agronomic trait determining plant architecture and yield; however, the molecular mechanisms underlying branching in the stalk vegetable, flowering Chinese cabbage, remain unclear. The present study identified two tandem genes responsible for primary rosette branching in flowering Chinese cabbage by GradedPool-Seq (GPS) combined with Kompetitive Allele Specific PCR (KASP) genotyping. A 900 kb candidate region was mapped in the 28.0−28.9 Mb interval of chromosome A07 through whole-genome sequencing of three graded-pool samples from the F_2_ population derived by crossing the branching and non-branching lines. KASP genotyping narrowed the candidate region to 24.6 kb. Two tandem genes, *BraA07g041560.3C* and *BraA07g041570.3C*, homologous to *AT1G78440* encoding GA2ox1 oxidase, were identified as the candidate genes. The *BraA07g041560.3C* sequence was identical between the branching and non-branching lines, but *BraA07g041570.3C* had a synonymous single nucleotide polymorphic (SNP) mutation in the first exon (290^th^ bp, A to G). In addition, an ERE *cis*-regulatory element was absent in the promoter of *BraA07g041560.3C*, and an MYB *cis*-regulatory element in the promoter of *BraA07g041570.3C* in the branching line. Gibberellic acid (GA_3_) treatment decreased the primary rosette branch number in the branching line, indicating the significant role of GA in regulating branching in flowering Chinese cabbage. These results provide valuable information for revealing the regulatory mechanisms of branching and contributing to the breeding programs of developing high-yielding species in flowering Chinese cabbage.

## Introduction

*Brassica rapa* is one of the most important *Brassica* species with a long cultivation history, has diverse and distinct morphological traits. Flowering Chinese cabbage [*Brassica rapa* L. ssp*. chinensis* (L.) Hanelt var. *parachinensis* (L.H. Bailey)] is that bolts readily (Wang and Kole et al., 2015). It is a stalk vegetable, and the stems with flower buds and leaves are consumed after cooking, especially in southern and central China and southeastern Asian countries. Typically, only one flowering stalk (main stem, without primary rosette branches) can be harvested from the common varieties, while several stalks are produced (12 stalks, primary rosette branches) on the local flowering Chinese cabbage variety named Zengcheng (Niu et al., 2019). Thus, the primary rosette branching trait influences plant architecture and yield in flowering Chinese cabbage. Therefore, identifying genes is important to develop varieties with multiple branches and meet the growing demand for flowering Chinese cabbage.

The activities of meristems, including apical, axillary and inflorescence meristems, basically determine the branching trait (Wang et al., 2018a). In flowering Chinese cabbage, the shoot apical meristem (SAM) turns into an inflorescence meristem that produces flowers directly or flower-bearing shoots after transitioning from the vegetative to the reproductive phase. The primary rosette branches grow from the axillary buds subtended by rosette leaves. Then, the secondary inflorescence branches grow at the axils of the cauline leaves on the elongated internodes of the main inflorescence stem, like that in *Arabidopsis thaliana* (Wang et al., 2018a; Fichtner et al., 2021). Studies in rice, *Arabidopsis*, and several other species characterizing the regulatory components of tiller or branch development have improved our understanding of branching (Wang and Li, 2011; Fichtner et al., 2021).

Typically, branching is a quantitative trait controlled by multiple genes and is susceptible to the environment (Ehrenreich et al., 2007; Kebrom et al., 2013) and plant hormones (Wang and Li, 2011). The hormonal control of bud outgrowth is complex and not yet fully understood. Auxin (Morris et al., 2005), cytokinin (Xu et al., 2015), strigolactone (De Jong et al., 2014), gibberellin (Martínez-Bello et al., 2015), abscisic acid (Holalu et al., 2020), and their interactions (Chen et al., 2013; Cao et al., 2017) have been reported to affect branching. Among these, gibberellin significantly influences the growth of branches and main stems. Okada et al. (2020) found that gibberellic acid (GA_3_) application increased the number of lateral branches on apple trees. Meanwhile, the exogenous spraying of GA_3_ rescued the dwarf phenotype of the legume’s *msd1-2* (multi-*seeded1-2*) mutant (Li et al., 2021). Researchers have identified a few genes controlling branching in *Brassica juncea, Brassica napus*, non-heading Chinese cabbage, and purple flowering Chinese cabbage (Li, 2018; Muntha et al., 2019; Li et al., 2020a; Li et al., 2020b). However, branching in flowering Chinese cabbage has not been fully clarified.

Researchers recently proposed a new quantitative trait mapping technique called GradedPool-Seq (GPS) for rapidly mapping the quantitative trait loci (QTL), it can rapidly identify QTLs for complex traits comparing conditional methods (Wang et al., 2019). GPS with high-throughput sequencing scores and assigns the F_2_ populations derived from a distant cross of parental lines exhibiting contrasting phenotypes into three or more graded groups based on phenotypic values. GPS has been successfully applied to dissect heterotic genes of thousand-grain weight, plant height, heading date, flag leaf angle, and tiller angle in rice. Moreover, the candidate intervals identified by the GPS method is consistent with the mapping interval obtained by the traditional method in rice (Wang et al., 2019).

Therefore, the present study used GPS with Kompetitive Allele Specific PCR (KASP) genotyping to map and identify the candidate genes associated with primary rosette branches in flowering Chinese cabbage. We further analyzed the similarities and variations in the full length and promoters of the candidate genes between the lines with different branching phenotypes. Finally, we investigated the role of GA_3_ in regulating the development of multiple primary rosette branches in flowering Chinese cabbage. The findings of our study will provide novel insights into the mechanisms of branching and lay a foundation for developing flowering Chinese cabbage cultivars with multiple primary rosette branches.

## Materials and methods

### Plant materials and growing conditions

‘CX020’ (Branching line), a doubled haploid (DH) line derived by microspore culture from Zengcheng flowering Chinese cabbage [*Brassica rapa* L. ssp*. chinensis* (L.) Hanelt var. *parachinensis* (L.H. Bailey)] with multiple primary rosette branches at harvest, and ‘CX010’ (Non-branching line), a DH line derived from Guangdong flowering Chinese cabbage with one flower-bearing shoot and no primary rosette branch, were used as parents in this study. These DH parents were crossed to generate the F_1_ and F_2_ populations for the phenotypic and genetic analyses. These DH parents exhibited stable inheritance after multiple seasons of planting. All the plants (‘CX010’, ‘CX020’, and F_2_ population) were grown at the Shenyang Agricultural University experimental base (Shenyang, China, *41°82′N, 123°24′E)* in 2019. The plants were sown on August 1st, and the number of primary rosette branches was analyzed on September 15th. The primary rosette branches were analyzed on the axillary branches subtended by rosette leaves 5 cm away from the cotyledonary node.

### GradedPool-Seq

Three types of pools (50 plants each), including ‘multiple primary rosette branching (12−15 branches)’, ‘less primary rosette branching (1−3 branches)’ and ‘moderate primary rosette branching (7−8 branches)’ plants were selected *via* phenotypic analysis from the F_2_ population, consisting of 1050 individuals, were used for GPS analysis with the two parents. Young and fresh leaves of the parents and the selected F_2_ individuals were harvested separately for total genomic DNA extraction using the Plant Genomic DNA Kit (Tiangen, Beijing, China), following the manufacturer’s instructions. The quantity and quality of the DNA were ensured using spectrophotometric analysis and 2% agarose gel electrophoresis. The DNA samples were quantified using a Qubit fluorometer and pooled at equimolar concentrations to generate the ‘multiple branching’, ‘less branching’ and ‘moderate branching’ pools separately. Pair-end sequencing (PEN150) on an Illumina Novaseq system (Illumina, USA) was performed following the standard protocol. The sequencing data from each hybrid pool was merged and aligned to the reference genome (http://brassicadb.org/brad/datasets/pub/Genomes/Brassica_rapa/V3.0/) to calculate the depth of each variant. After filtering the variants with low quality and depth using the default parameters, Ridit analysis was performed to calculate the p-value for each variant. The sliding window size was set to 0.2 Mb, the threshold p-value to 10^-8^, and the candidate regions to peak intervals to reduce background noise and identify the significant variants.

### KASP genotyping

KASP was performed on a high-throughput Intelliqube genotyping platform for genotyping of the F_2_ individuals. DNA was extracted from 150 F_2_ individuals and the parents using the CTAB method. The DNA concentration and quality were assessed on a BioDropuLite microanalyzer (BioDrop, Britain), and the samples were diluted to a suitable concentration (5−10 ng/μL). KASP primers (**Supplementary Table S1**) were designed for 19 SNP loci (**Supplementary Table S2**) using Primer Premier 5.0 (Singh et al., 1998), and KASP assays were conducted in a 384-well plate format on a Hydrocycler (LGC, Middlesex, UK) using the following PCR protocol: 94 °C for 15 min; 94 °C for 20 s, 61 °C for 60 s (-1 °C/cycle, 10 cycles in total), and 94 °C for 20 s; 55 °C for 60 s (26 cycles). The components of the KASP reaction mixture are shown in **Supplementary Table S3**. The fluorescence signal generated was measured on an IntelliQube (LGC, Middlesex, UK). Finally, based on the KASP genotypic data and phenotypic data of the F_2_ individuals, the QTL IciMapping software v4.2 (Liu et al., 2019) generated the linkage map to obtain the QTL and further narrow down the candidate interval.

### Candidate gene analysis

Gene annotation information of the target region was obtained from the *Brassica* database (http://brassicadb.org/brad/index.php) and the *Arabidopsis* database (https://www.arabidopsis.org/). The candidate genes’ full-length sequence and a 2000 bp long promoter sequence were amplified with the specific primers using PCR, and the amplicons were purified using a Gel Extraction Kit (CWBIO, Beijing, China). The purified products were introduced into the pGEM^®^-T Easy Vector (Promega, USA) and transformed into Top10 competent cells (CWBIO, Beijing, China). The colonies were sequenced at Sangon Biotech (Shanghai, China), and the sequences were aligned using DNAMAN 6.0 (Lynnon Biosoft, Canada). Meanwhile, PlantCARE (http://bioinformatics.psb.ugent.be/webtools/plantcare/html/) was used to predict the *cis*-regulatory elements in the promoter regions of the candidate genes.

### Expression analysis of the candidate genes

Total RNA was extracted from the whole roots, rosette stems, tender cauline leaves, flowers of top inflorescences and shoot tips of the parents. The rosette stem of CX020 at five different stages (every ten days), the first sample was taken when CX020 was in the fourth euphylla stage. The RNA was reverse transcribed using the FastKing RT Kit (Tiangen, Beijing). Real-time quantitative reverse transcription PCR (qRT-PCR) was carried out to determine the expression levels of the candidate genes using cDNA as the template with the SYBR Green PCR Master Mix on QuantStudio™ 6 Flex (Applied Biosystems, USA), maintaining three biological replicates per sample. The *Actin* gene was used as an internal reference control. The primers used for qRT-PCR were as follows: *BraA07g041560.3C* (F:5^′^-TGGAGATGATTACTGATGGGTTA-3^′^; R: 5^′^-ATTTTCGTGGATGAGAGGGC-3^′^); *BraA07g041570.3C* (F:5^′^-TCCTGGATTTCTGTCCCTTC-3^′^; R: 5^′^-ACCCTATGCTTCACGCTTTT-3^′^); *Actin* (F: 5^′^- ATCTACGAGGGTTATGCT-3^′^; R: 5^′^-CCACTGAGGACGATGTTT -3^′^). The relative gene expression levels were calculated following the 2^−ΔΔCt^ method.

### Exogenous GA_3_ and PAC treatment

We predicted that the genes affecting branching might be related to GA_3_. An experiment was carried out by spraying GA_3_ (750 mg/L) on the multiple primary rosette branching parent line CX020, and spraying Paclobutrazol (PAC, 0.3×10^-3^ mg/L on the non-primary rosette branching parent line CX010, using water as a control. The plants were sown on August 1st in green house at Shenyang Agricultural University under long-day conditions and GA_3_ treatment was carried out on the fourth euphylla stage (August 15th); spraying was carried out every two days until phenotypes of fewer branches appeared. Twenty plants were maintained per treatment, using three biological replicates. The primary rosette branches number was recorded when the branching phenotype is obvious.

## Results

### Phenotypic characterization of primary rosette branching in flowering Chinese cabbage

**Figure 1:**
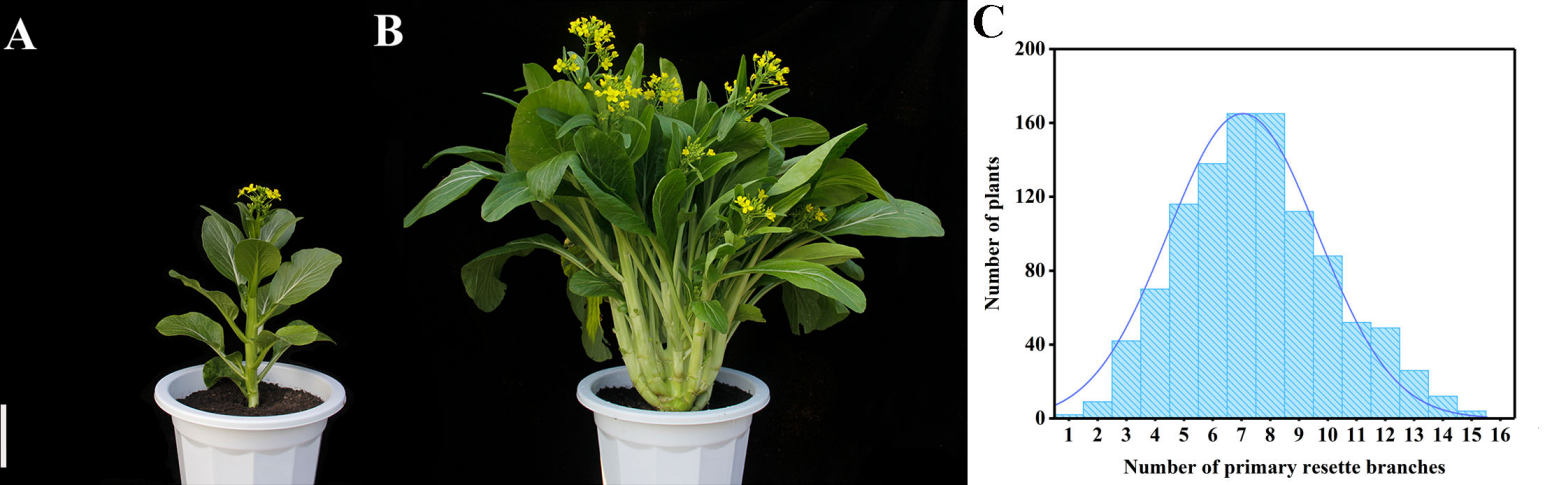
Phenotype of parent lines CX010, CX020 and distribution of the number of primary rosette branches in flowering Chinese cabbage. **(A)** Non-primary rosette branching line (‘CX010’). **(B)** Multiple primary rosette branching line (‘CX020’) Bar = 10 cm. **(C)** Distribution of the number of primary rosette branches in 1050 individuals of F_2_ population.

During the reproductive growth stage, ‘CX010’ (parent 1, **Figure 1A**) had only one main stalk but no primary rosette branches on the rosette stem, while ‘CX020’ (parent 2, **Figure 1B**) had 14 primary rosette branches that contributed to the yield. Moreover, significant differences were observed in the number of primary rosette branches between the non-branching line (‘CX010’) and the multiple branching line (‘CX020’). Phenotypic segregation analysis showed that F_1_ progeny had a moderate number of primary rosette branches. The number of primary rosette branches in the 1050 F_2_ plants ranged from 0 to 15 and showed a normal distribution (**Figure 1C**). These observations indicated the role of QTLs in controlling the number of primary rosette branching in flowering Chinese cabbage.

### Candidate region for primary rosette branching identified by GPS

**Figure 2:**
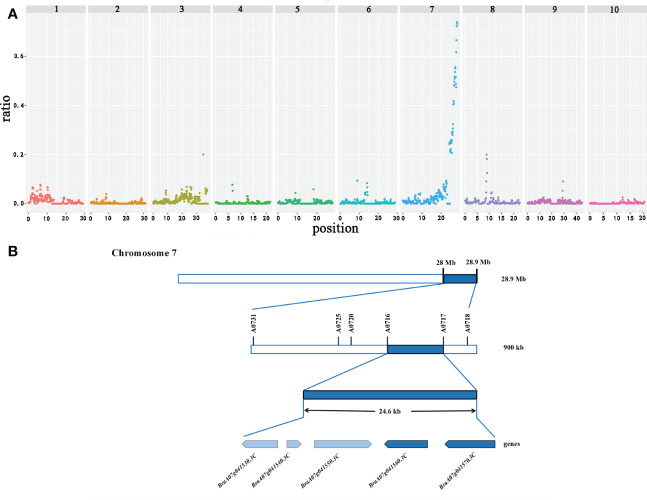
Candidate interval and genes identified by GPS on chromosome A07 in flowering Chinese cabbage. **(A)** The X-axis value is set at a midpoint at each defined genomic interval, and the Y-axis value corresponds to the ratio. **(B)** Candidate genes identified in the targeted interval.

The sequences obtained from the three pools (12−15 branches, 7−8 branches, and 1−3 branches) were mapped to the reference genome (http://brassicadb.org/brad/datasets/pub/Genomes/Brassica_rapaV3.0/) to estimate the allelic frequencies. Ridit analysis was implemented with allelic frequencies from three bulks to calculate p-values for each SNP. The background noise complicated the precise localization of the QTLs. Subsequently, the statistical noise-reducing strategy narrowed the interval to about 900 kb (28.0−28.9 Mb; significant peak) on chromosome A07, which was identified as the main QTL controlling the primary rosette branching. A few minor peaks also appeared on the other chromosomes that may be minor QTLs (**Figure 2A**). However, we further focused on the main QTL.

### Further mapping of the primary rosette branching gene

A total of 178 genes were identified in the candidate region based on the gene information in the *Brassica* database (**Supplementary Table S4**). Then, to locate the candidate genes controlling the primary rosette branching in this region, we developed 18 KASP markers according to SNPs in this region. The genotype of 150 F_2_ individuals and two parents was detected. Only one QTL was discovered associated with the number of primary rosette branches in the F_2_ based on KASP, which accounted for 20.81% of the phenotypic variance. Markers A0716 and A0717, located at the two sides of the candidate gene, were the most closely associated with the candidate gene, and the physical distance between these markers was 24.6 kb, which contained five genes (**Figure 2B**).

### Identification of the candidate genes related to primary rosette branching

Then, all the candidate genes in the candidate region were analyzed based on the *Brassica* database to identify the candidate genes that influence primary rosette branching. To further identify the candidate genes that influence primary rosette branching, five annotated genes in the candidate region were analyzed (**Table 1**). Among these, two tandem genes, *BraA07g041560.3C* and *BraA07g041570.3C*, were found homologous to the *Arabidopsis thaliana AT1G78440*, which encodes gibberellin 2-oxidase that acts on C19 gibberellins. In rice, *GA2oxs* influence the number of tillers (Lo et al., 2008). Therefore, we hypothesized that these two tandem genes might be associated with the number of primary rosette branches in flowering Chinese cabbage.

**Table 1:** Annotation of the genes within the mapped region on chromosome A07 in flowering Chinese cabbage.

Gene	Start	End	Gene annotations (BLASTX to *Arabidopsis thaliana*)	E value
*BraA07g041530.3C*	28005077	28006447	PGX2 is a cell wall protein that codes for a polygalacturonase.	0.0
*BraA07g041540.3C*	28013792	28014106	VQ motif-containing protein; (source: Araport11)	1.72^e-51^
*BraA07g041550.3C*	28018304	28020467	Activates the latent peptidases DA1, DAR1 and DAR2 by mono-ubiquitination at multiple sites. Subsequently, these activated peptidases destabilize various positive regulators of growth.	5.64^e-45^
*BraA07g041560.3C*	28023878	28025430	Encodes a gibberellin 2-oxidase that acts on C19 gibberellins.	0.0
*BraA07g041570.3C*	28036581	28038308	Encodes a gibberellin 2-oxidase that acts on C19 gibberellins.	0.0

### Candidate gene cloning and sequence analysis

**Figure 3:**
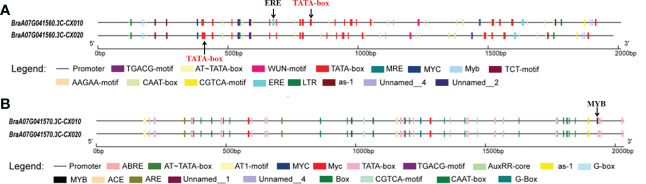
The *cis*-regulatory elements analysis of *BraA07g041560.3C* and *BraA07g041570.3C* promoters in parents CX010 and CX020. **(A)** Analysis of *BraA07g041560.3C* gene promoters. **(B)** Analysis of *BraA07g041570.3C* gene promoters.

These two full-length genes (*BraA07g041560.3C* and *BraA07g041570.3C*) were cloned to analyze the variances between the parents (**Supplementary Figures S1**, **S2**). Sequence analysis revealed that the full-length sequence of *BraA07g041560.3C* was identical in the parents, whereas *BraA07g041570.3C* had a synonymous SNP mutation from A to G at the 290^th^ position (**Supplementary Figure S2**). The promoter sequence of *BraA07g041560.3C* had a 31 bp deletion in CX020 (**Supplementary Figure S3**). There were many differences of the promoter sequence in *BraA07g041560.3C* between CX020 and CX010 that resulted in the absence of an ERE *cis*-regulatory element and the position changes of many cis-acting elements in CX020 (**Supplementary Figure S3**; **Figure 3A**). Compared to CX010, CX020 had an SNP in the promoter of *BraA07g041570.3C*, 103 bp upstream of the translation initiation site, resulting in the absence of an MYB *cis*-regulatory element (**Supplementary Figure S4**; **Figure 3B**).

### Expression analysis of candidate genes

**Figure 4:**
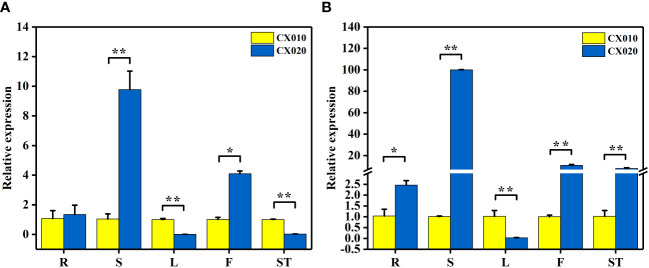
Expression levels of *BraA07g041560.3C* and *BraA07g041570.3C* in different tissues of CX010 and CX020 plants based on qRT-PCR. **(A)** The expression level of *BraA07g041560.3C.* **(B)** The expression level of *BraA07g041570.3C.* R, whole roots; S, rosette stems; L, tender cauline leaves; F, flowers of top inflorescences; ST, shoot tips. The data shown are the means of three replicates (± SD). * and ** indicate significant differences in expression levels at P < 0.05 and P < 0.01, respectively (Student’s *t*-test).

**Figure 5:**
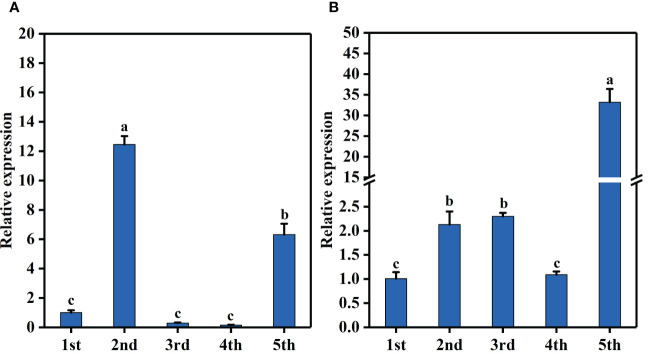
Expression levels of *BraA07g041560.3C* and *BraA07g041570.3C* in CX020 rosette stem at different stages based on qRT-PCR. **(A)** The expression level of *BraA07g041560.3C.* **(B)** The expression level of *BraA07g041570.3C.* The data shown are the means of three replicates (± SD). The different lowercase letters above the means are significantly different at P=0.05 level.

Further, qRT-PCR was used to detect the relative expression levels of *BraA07g041560.3C* and *BraA07g041570.3C* in CX010 and CX020. The relative expression level of *BraA07g041560.3C* in the stem and flower of CX020 was significantly higher than that in CX010, while that in the leaf and shoot tip of CX020 was markedly lower than that in CX010 (**Figure 4A**). The relative expression level of *BraA07g041570.3C* in CX020 was substantially higher in root, stem, flower, and shoot tip than that of CX010 but significantly lower in the leaf (**Figure 4B**). We further analyzed the differences in the expression levels of *BraA07g041560.3C* and *BraA07g041570.3C* in CX020 stem at different stages. The analysis revealed that the *BraA07g041560.3C* expression was the highest at the second stage (**Figure 5A**). Meanwhile, the *BraA07g041570.3C* expression level at the last stage was significantly different from those at the other stages (**Figure 5B**).

### Phenotypic features after GA_3_ and PAC treatment

**Figure 6:**
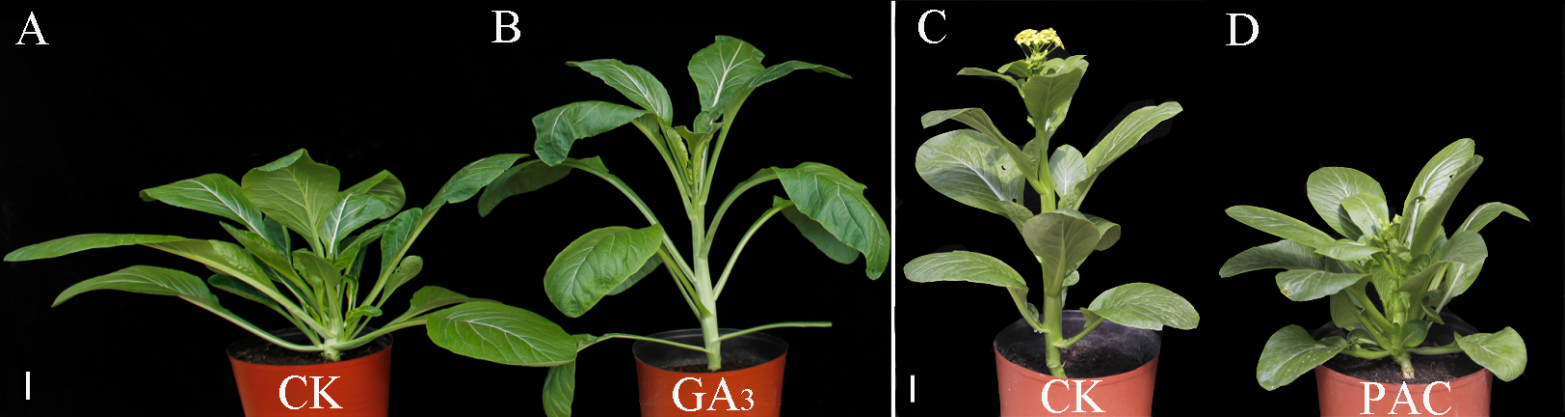
Phenotypes of CX020 and CX010 after treatments with exogenous GA_3_ and PAC. **(A, B)** The CK and exogenous GA_3_ treatment of CX020. Bar = 8 cm. **(C, D)** The CK and exogenous PAC treatment of CX010. Bar = 5 cm.

**Figure 7:**
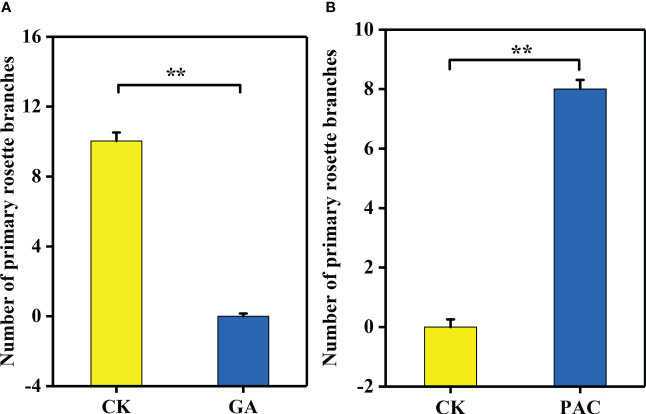
The number of primary rosette branches of CX020 and CX010 treated with exogenous GA_3_ and PAC. **(A)** The CK and exogenous GA_3_ treatment of CX020. **(B)** The CK and exogenous PAC treatment of CX010. The data shown are the means of three replicates (± SD). ** indicate significant differences in expression levels at P < 0.01 (Student’s t-test).

GA_3_ treatment significantly decreased the number of primary rosette branches (**Figures 6A, B**). On the contrary, after PAC treatment on the non-branching line CX010, the number of primary rosette branches were significantly increased (**Figures 6C, D**). The number of rosette branches significantly decreased from 10 to 0 after GA_3_ treatment (**Figure 7A**), while the number of rosette branches significantly increased from 0 to 8 after PAC treatment (**Figure 7B**).

## Discussion

Branching is an important trait that determines plant architecture, directly influences yield, and is closely related to environmental adaptation (Teichmann and Muhr, 2015; Mathan et al., 2016). The primary rosette branches that contribute to yield in flowering Chinese cabbage are different from the rosette branches in non-heading Chinese cabbage and tillers in cereal crops. In non-heading Chinese cabbage, basal branches arise from axillary meristems in the leaf axils subtended by rosette leaves during the vegetative stage (Cao et al., 2016). Then the inflorescence branches grow out from the axillary meristems. In cereal crops, tillers arise from non-elongated internodes at the base of the parent shoot during the vegetative growth phase and survive even if the primary shoot dies because tillers generally produce adventitious roots (Kebrom et al., 2013). While, the branches in flowering Chinese cabbage develop only after transitioning to the reproductive phase, the stage at which tiller development ceases in cereal crops, such as wheat, barley, and rice. Studies have identified genes related to branching in *Brassica* crops. Li et al. predicted *BnaA09.ELP6* controls effective primary cauline branching (arising from the main stem) in *Brassica napus* (Li et al., 2020a). Meanwhile, shoot branching in non-heading Chinese cabbage (*Brassica rapa* ssp. *chinensis* Makino) is controlled by *BrSB9.1* (*Bra007056*), which is homologous to *MOC1* that controls tillering in rice (Li et al., 2020b). *Bra004212*, the homolog of *TCP1*, was identified as the candidate gene for tillering in purple flowering Chinese cabbage (Li, 2018). *PAT1* (Phytochrome A signal transduction 1), which belongs to the GRAS transcription factor family, negatively regulates branching in leafy *Brassica juncea* (Muntha et al., 2019). However, the genes controlling primary rosette branching in Chinese cabbage differ from these reported species. The present study the first time identified for two tandem genes, *BraA07g041560.3C* and *BraA07g041570.3C*, which are homologous to *AT1G78440* encoding *GA2ox1* oxidase, as potential candidate genes responsible for primary rosette branching.

Traditional QTL mapping using genetic map construction by traditional markers and phenotyping is a reliable approach to isolate genes or QTLs associated with agronomic traits (Li et al., 2020b). Hundreds of SSR (Simple Sequence Repeat), InDels, or SLAF (Specific-Locus Fragment)markers have been developed to construct a primary genetic linkage map based on the population of the F_2_ generation, doubled haploid (DH) or recombinant inbred line (RIL) and locate QTL according to the phenotype (Liu et al., 2019). Usually, to narrow down the region and screen for a few candidate genes, a near-isogenic line (NIL) population is needed. However, the process of developing the NIL population is time-consuming and labor-intensive. GPS is a quick and efficient method to ascertain the genomic regions that harbor QTL for complex quantitative traits (Wang et al., 2019). It accelerates gene mapping by sequencing the graded pools; here, only the F_2_ population is needed. Bulked-segregant analysis (BSA) also rapidly and effectively locates genes by constructing segregating F_2_ populations from parents with significant phenotypic differences and selecting individuals with extreme traits to build a DNA pool for sequencing (Giovannoni et al., 1991; Michelmore et al., 1991; Takagi et al., 2013; Zou et al., 2016). However, the GPS has a higher resolution (~400 kb in rice) (Wang et al., 2019) than BSA (3 Mb) (Wang et al., 2018b; Liu et al., 2019; Yang et al., 2021). GPS identified a QTL controlling fruit size in melons, and traditional QTL mapping validated the results (Lian et al., 2021). Liu et al. (2020) successfully identified two candidate intervals controlling extremely late flowering in rice by GPS. In this work, one major candidate region associated with primary rosette branching was finally located in a 900 kb region on chromosome A07 based on three graded pools according to the primary rosette branching number of the F_2_ population in flowering Chinese cabbage, with 178 genes. To our knowledge, this is the first report on the genetic control of primary rosette branching in flowering Chinese cabbage using GPS.

KASP genotyping based on uniplex SNP is a novel approach to fine map genes using F_2_ individuals combined with their phenotype data (Xu et al., 2018; Cheng et al., 2021). Liu et al. (2019) narrowed the candidate region from 3.29 Mb to 790 kb by QTL analysis using KASP markers with 147 F_2_ individuals in tomatoes and identified the *Cf*-*10* gene (*Cladosporium fulvum*). Lei et al. (2020) narrowed the genome interval from 4.17 Mb to 222 kb by 26 KASP markers genotyping with 199 individuals randomly selected from F_2:3_ and identified a major QTL and candidate gene for salt tolerance in rice. In this study, we genotyped 150 F_2_ individuals using 18 KASP markers and narrowed down the candidate region from 900 kb to 24.6 kb, with five genes.

GA2 oxidases are dioxygenases encoded by multiple genes and the key enzymes involved in gibberellin metabolism (Hedden and Phillips, 2000). GA2 oxidase typically transforms the bioactive GA1 and GA4 into the inactive catabolic metabolites GA8 and GA34, respectively, reducing the activity of GA and maintaining the balance between bioactive and inactive GA (Claeys et al., 2014). Studies have demonstrated the role of *GA2ox1* in regulating rosette branching in some crops. Higher expression levels of *GA2ox* genes have been correlated to low concentrations of bioactive GAs (Schomburg et al., 2003; Dijkstra et al., 2008; Zhou et al., 2011). Meanwhile, turfgrass (*Paspalum notatum* Flugge) overexpressing *AtGA2ox1* had significantly lower levels of active GA but more tillers than wild-type plants (Agharkar et al., 2007). Overexpression of the *OsGA2ox* in rice increased tiller number (Lo et al., 2008), consistent with an increased number of tillers observed with the overexpression of *PvGA2ox5* and *PvGA2ox9* in switchgrass (*Panicum virgatum* L.) (Wuddineh et al., 2014). On the other hand, silencing of five *GA2ox* genes in tomatoes significantly increased GA4 content and inhibited lateral branches (Martínez-Bello et al., 2015). Therefore, we speculated that the two tandem genes, *BraA07g041560.3C* and *BraA07g041570.3C*, homologous to the *Arabidopsis thaliana* gene (*AT1G78440*) encoding a gibberellin 2-oxidase, found in the candidate region might be associated with primary rosette branching in flowering Chinese cabbage. While in the candicate region, the other three genes were not found to be related to branching. *BraA07g041530.3C* was the homologs gene of *AtPGX2*, which has been demonstrated regulating root hair development in response to phospho-starved (Zhang et al., 2022). *BraA07g041540.3C* was the homologs gene of *AtVQ10*, which has been shown to interact with *WRKY33* to affect plant sizes at mature stages in *Arabidopsis* (Cheng et al., 2012). *BraA07g041550.3C* was found to be homologous to *Arabidopsis At1g78420* (*DA2*), which encoded RING-type protein with E3 ubiquitin ligase activity and regulated seed size by restricting cell proliferation in the maternal integuments of developing seeds (Xia et al., 2013).

Typically, variations in the promoter regions may lead to changes in gene expression levels (Mito et al., 1996). The expression levels of these two genes were significantly higher in rosette stems of the branching line than in the non-branching line. Detailed analysis revealed differences in the promoter sequences between the parents. The promoter of the *BraA07g041560.3C* gene missed an ERE *cis*-regulatory element in branching line CX020, with a difference in the location of a TATA-box and the *cis*-regulatory element. However, there is no evidence for the role of the ERE *cis*-regulatory element in the formation of branching. Meanwhile, the promoter sequence of the *BraA07g041570.3C* gene lacked an MYB *cis*-regulatory element in CX020. Studies presented associated several genes belonging to the MYB family (MYB2, MYB37, and MYB181) with axillary meristem development and branching in *Arabidopsis* (Guo and Gan, 2011; Keller et al., 2006; Yang et al., 2018). AtMYB2 protein represses the formation of axillary meristems in response to salt and drought stresses (Jia et al., 2020). Meanwhile, the overexpression of *GmMYB181* in *Arabidopsis* altered the plant architecture, increased lateral branches, and reduced plant height (Yang et al., 2018). Therefore, we hypothesized that the *MYB* gene control primary rosette branching in flowering Chinese cabbage.

GAs are a large group of diterpenoid natural products characterized by tetracyclic 6-5-6-5 ring derived from ent-gibberellane (MacMillan and Takahashi, 1968; Peters, 2010). The biosynthesis of GA is a complex multi-step process requiring a variety of functional enzymes to catalyze the different intermediates (Wei et al., 2019). GA normally inhibits shoot branching, and plants overexpressing GA catabolic genes and GA-deficient mutants exhibit more shoot branching comparing to the wild-type (Silverstone et al., 1997; Agharkar et al., 2007; Lo et al., 2008). GA regulates internode elongation in rice, where the bioactive GA probably prevents from reaching the nodes below the shoot apex and inhibits internode elongation during the vegetative phase (Sakamoto et al., 2001).In this work, exogenous GA_3_ application significantly reduced the rosette branches of flowering Chinese cabbage.

## Conclusions

The present study identified two tandem genes, *BraA07g041560.3C* and *BraA07g041560.3C*, homologous to *AT1G78440* encoding GA2ox1 oxidase, as the candidates genes related to primary rosette branching in flowering Chinese cabbage. We detected differences in *cis*-regulatory elements in the promoter sequences of *BraA07g041560.3C* and *BraA07g041570.3C* between the branching and non-branching lines, which indicated the role of genes in regulating branching. These results provide valuable information for revealing the branching regulatory mechanisms in flowering Chinese cabbage. Further studies should investigate and conform the possible function and the promoter activity of *BraA07g041560.3C* and *BraA07g041570.3C* in flowering Chinese cabbage.

## Data availability statement

The data presented in the study are deposited in the SRA repository of the National Center for Biotechnology Information, accession number PRJNA908111 (https://www.ncbi.nlm.nih.gov/bioproject/PRJNA908111.

## Ethics statement

The authors note that this research was performed and reported in accordance with ethical standards of scientific conduct.

## Author contributions

YZ and HF designed the experiments. JG and JL conducted the experiments and wrote the manuscript. JG, JL, and QY performed the data analysis. YZ revised the manuscript. All authors reviewed and approved this manuscript.

## Supplementary material

Supplementary Figure 1 The full-length of gene *BraA07g041560.3C*.

Supplementary Figure 2 The full-length of gene *BraA07g041570.3C*.

Supplementary Figure 3 The promoter sequence of *BraA07g041560.3C*.

Supplementary Figure 4 The promoter sequence of *BraA07g041570.3C*.

Supplementary Table 1 KASP primer sequence used in this study.

Supplementary Table 2 SNP site information.

Supplementary Table 3 The components of the KASP reaction mixture.

Supplementary Table 4 The 178 genes identified in the candidate region based on the gene information in the *Brassica* database.

## References

[B1] Agharkar M. Lomba P. Altpeter F. Zhang H. N. Kenworthy K. Lange T. (2007). Stable expression of *AtGA2ox1* in a low-input turfgrass (*Paspalum notatum* flugge) reduces bioactive gibberellin levels and improves turf quality under field conditions. Plant Biotechnol. J. 5, 791–801. doi: 10.1111/j.1467-7652.2007.00284.x 17764521

[B2] Cao X. W. Cui H. M. Yao Y. Xiong A. S. Hou X. L. Li Y. (2017). Effects of endogenous hormones on variation of shoot branching in a variety of non-heading Chinese cabbage and related gene expression. J. Plant Biol. 60, 343–351. doi: 10.1007/s12374-016-0124-2

[B3] Cao X. W. Cui H. M. Li J. (2016). Heritability and gene effects for tiller number and leaf number in non-heading Chinese cabbage using joint segregation analysis. Scientia Horticulturae 203, 199–206. doi: 10.1016/j.scienta.2016.03.018

[B4] Cheng Z. K. Liu Z. G. Xu Y. C. Ma L. L. Chen J. Y. Gou J. Q.. (2021). Fine mapping and identification of the candidate gene *BFS* for fruit shape in wax gourd (*Benincasa hispida*). Theor. Appl. Genet. 134, 3983–3995. doi: 10.1007/s00122-021-03942-8 34480584

[B5] Cheng Y. Zhou Y. Yang Y. (2012). Structural and functional analysis of VQ motif-containing proteins in arabidopsis as interacting proteins of WRKY transcription factors. Plant Physiol. (Rockville) 159 (2), 810. doi: 10.1104/pp.112.196816 22535423 PMC3375943

[B6] Chen X. L. Zhou X. Y. Xi L. Li J. X. Zhao R. Y. Ma N.. (2013). Roles of *DgBRC1* in regulation of lateral branching in chrysanthemum (*Dendranthema* x *grandiflora* cv. jinba). PLoS One 8, e61717. doi: 10.1371/journal.pone.0061717 23613914 PMC3629106

[B7] Claeys H. De Bodt S. Inzé D. (2014). Gibberellins and DELLAs: central nodes in growth regulatory networks. Trends Plant Sci. 19, 231–239. doi: 10.1016/j.tplants.2013.10.001 24182663

[B8] De Jong M. George G. Ongaro V. Williamson L. Willetts B. Ljung K.. (2014). Auxin and strigolactone signaling are required for modulation of Arabidopsis shoot branching by nitrogen supply. Plant Physiol. 166, 384–395. doi: 10.1104/pp.114.242388 25059707 PMC4149722

[B9] Dijkstra C. Adams E. Bhattacharya A. Page A. F. Anthony P. Kouurmptli S.. (2008). Over-expression of a gibberellin 2-oxidase gene from *Phaseolus coccineus* L. enhances gibberellin inactivation and induces dwarfism in *Solanum* species. Plant Cell Rep. 27, 463–470. doi: 10.1007/s00299-007-0471-z 17999064

[B10] Ehrenreich I. M. Stafford P. A. Purugganan M. D. (2007). The genetic architecture of shoot branching in *Arabidopsis thaliana*: A comparative assessment of candidate gene associations vs. quantitative trait locus mapping. Genetics 176, 1223–1236. doi: 10.1534/genetics.107.071928 17435248 PMC1894586

[B11] Fichtner F. Barbier F. F. Annunziata M. G. Feil R. Olas J. J. Mueller-Roeber B.. (2021). Regulation of shoot branching in *Arabidopsis* by trehalose 6-phosphate. New Phytol. 229, 2135–2151. doi: 10.1111/nph.17006 33068448

[B12] Giovannoni J. J. Wing R. A. Ganal M. W. Tanksley S. D. (1991). Isolation of molecular markers from specific chromosomal intervals using DNA pools from existing mapping populations. Nucleic Acids Res. 19, 6553–6558. doi: 10.1093/nar/19.23.6553 1684420 PMC329217

[B13] Guo Y. F. Gan S. S. (2011). *AtMYB2* regulates whole plant senescence by inhibiting cytokinin-mediated branching at late stages of development in *Arabidopsis* . Plant Physiol. 156, 1612–1619. doi: 10.1104/pp.111.177022 21543729 PMC3135943

[B14] Hedden P. Phillips A. L. (2000). Gibberellin metabolism: new insights revealed by the genes. Trends Plant Sci. 5, 523–530. doi: 10.1016/S1360-1385(00)01790-8 11120474

[B15] Holalu S. V. Reddy S. K. Blackman B. K. Finlayson S. A. (2020). Phytochrome interacting factors 4 and 5 regulate axillary branching *via* bud abscisic acid and stem auxin signalling. Plant,Cell Environ. 43, 2224–2238. doi: 10.1111/pce.13824 32542798

[B16] Jia T. Zhang K. Li F. Huang Y. F. Fan M. M. Huang T.. (2020). The AtMYB2 inhibits the formation of axillary meristem in Arabidopsis by repressing RAX1 gene under environmental stresses. Plant Cell Rep 39, 1755–1765. doi: 10.1007/s00299-020-02602-3 32970176

[B17] Kebrom T. H. Spielmeyer W. Finnegan E. J. (2013). Grasses provide new insights into regulation of shoot branching. Trends Plant Sci. 18, 41–48. doi: 10.1016/j.tplants.2012.07.001 22858267

[B18] Keller T. Abbott J. Moritz T. Doerner P. (2006). *Arabidopsis REGULATOR OF AXILLARY MERISTEMS1* controls a leaf axil stem cell niche and modulates vegetative development. Plant Cell 18, 598–611. doi: 10.1105/tpc.105.038588 16473968 PMC1383636

[B19] Lei L. Zheng H. L. Bi Y. L. Yang L. M. Liu H. L. Wang J. G.. (2020). Identification of a major QTL and candidate gene analysis of salt tolerance at the bud burst stage in rice (*Oryza sativa* l.) using QTL-seq and RNA-seq. Rice 13, 22. doi: 10.1186/s12284-020-00416-1 32778977 PMC7417472

[B20] Li Y. X. (2018). Genetic analysis and QTL mapping of tillering in purple caitai (Wuhan(HB: Huazhong Agricultural University).

[B21] Lian Q. Fu Q. S. Xu Y. Y. Hu Z. C. Zheng J. Zhang A. A.. (2021). QTLs and candidate genes analyses for fruit size under domestication and differentiation in melon (*Cucumis melo* l.) based on high resolution maps. BMC Plant Biol. 21, 126. doi: 10.1186/s12870-021-02904-y 33658004 PMC7931605

[B22] Li B. Gao J. Chen J. Wang Z. Shen W. Yi B.. (2020a). Identification and fine mapping of a major locus controlling branching in *Brassica napus* . Theor. Appl. Genet. 133, 771–783. doi: 10.1007/s00122-019-03506-x 31844964

[B23] Li W. Ma Q. Yin P. Wen J. Pei Y. Niu L.. (2021). The GA 20-oxidase encoding gene *MSD1* controls the main stem elongation in medicago truncatula. Front. Plant Sci. 12. doi: 10.3389/fpls.2021.709625 PMC8371406 34421956

[B24] Li P. Su T. Zhang B. Li P. Xin X. Yue X.. (2020b). Identification and fine mapping of *qSB.A09*, a major QTL that controls shoot branching in brassica rapa ssp. *chinensis* makino. Theor. Appl. Genet. 133, 1055–1068. doi: 10.1007/s00122-020-03531-1 31919538

[B25] Liu J. Gong J. Y. Wei X. Yang S. H. Huang X. H. Li C.. (2020). Dominance complementation of *Hd1* and *Ghd8* contributes to extremely late flowering in two rice hybrids. Mol. Breed. 40, 76. doi: 10.1007/s11032-020-01162-4

[B26] Liu G. Zhao T. You X. Q. Jiang J. B. Li J. F. Xu X. Y. (2019). Molecular mapping of the *Cf-10* gene by combining SNP/InDel-index and linkage analysis in tomato (*Solanum lycopersicum*). BMC Plant Biol. 19, 15. doi: 10.1186/s12870-018-1616-7 30621598 PMC6325758

[B27] Lo S. F. Yang S. Y. Chen K. T. Hsing Y. I. Zeevaart J. A. Chen L. J.. (2008). A novel class of gibberellin 2-oxidases control semidwarfism, tillering, and root development in rice. Plant Cell 20, 2603–2618. doi: 10.1105/tpc.108.060913 18952778 PMC2590730

[B28] MacMillan J. Takahashi N. (1968). Proposed procedure for the allocation of trivial names to the gibberellins. Nature 217, 170–171. doi: 10.1038/217170a0 5638147

[B29] Martínez-Bello L. Moritz T. López-Díaz I. (2015). Silencing C_19_-GA 2-oxidases induces parthenocarpic development and inhibits lateral branching in tomato plants. J. Exp. Bot. 66, 5897–5910. doi: 10.1093/jxb/erv300 26093022 PMC4566981

[B30] Mathan J. Bhattacharya J. Ranjan A. (2016). Enhancing crop yield by optimizing plant developmental features. Development 143, 3283–3294. doi: 10.1242/dev.134072 27624833

[B31] Michelmore R. W. Paran I. Kesseli R. V. (1991). Identification of markers linked to disease-resistance genes by bulked segregant analysis: a rapid method to detect markers in specific genomic regions by using segregating populations. Proceedings of the National Academy of Sciences 88, 9828–9832. doi: 10.1073/pnas.88.21.9828 PMC52814 1682921

[B32] Mito N. Wimmers L. Bennett A. (1996). Sugar regulates mRNA abundance of h+-ATPase gene family members in tomato. Plant Physiol. 112, 1229–1236. doi: 10.1104/pp.112.3.1229 8938420 PMC158050

[B33] Morris S. E. Cox M. C. Ross J. J. Krisantini S. Beveridge C. A. (2005). Auxin dynamics after decapitation are not correlated with the initial growth of axillary buds. Plant Physiol. 138, 1665–1672. doi: 10.1104/pp.104.058743 15965021 PMC1176436

[B34] Muntha S. T. Zhang L. Zhou Y. Zhao X. Hu Z. Yang J.. (2019). Phytochrome a signal transduction 1 and CONSTANS-LIKE 13 coordinately orchestrate shoot branching and flowering in leafy *Brassica juncea* . Plant Biotechnol. J. 17, 1333–1343. doi: 10.1111/pbi.13057 30578711 PMC6576096

[B35] Niu L. J. Shi F. Y. Feng H. Zhang Y. (2019). Efficient doubled haploid production in microspore culture of zengcheng flowering Chinese cabbage (*Brassica campestris* L. ssp. *Chinensis* [L.] makino var. *utilis* tsen et Lee). Scientia Hortic. 245, 57–64. doi: 10.1016/j.scienta.2018.09.076

[B36] Okada K. Wada M. Takebayashi Y. Kojima M. Sakakibara H. Nakayasu M.. (2020). Columnar growth phenotype in apple results from gibberellin deficiency by ectopic expression of a dioxygenase gene. Tree Physiol. 40, 1205–1216. doi: 10.1093/treephys/tpaa049 32333787

[B37] Peters R. J. (2010). Two rings in them all: The labdane-related diterpenoids. Natural Product Rep. 27, 1521–1530. doi: 10.1039/C0NP00019A PMC3766046 20890488

[B38] Sakamoto T. Kobayashi M. Itoh H. Tagiri A. Kayano T. Tanaka H.. (2001). Expression of a gibberellin 2-oxidase gene around the shoot apex is related to phase transition in rice. Plant Physiol. 125, 1508–1516. doi: 10.1104/pp.125.3.1508 11244129 PMC65628

[B39] Schomburg F. M. Bizzell C. M. Lee D. J. Zeevaart J. A. Amasino R. M. (2003). Overexpression of a novel class of gibberellin 2-oxidases decreases gibberellin levels and creates dwarf plants. Plant Cell 15, 151–163. doi: 10.1105/tpc.005975 12509528 PMC143488

[B40] Silverstone A. L. Mak PiuYing A. Casamitjana Martinez E. Sun T. (1997). The new RGA locus encodes a negative regulator of gibberellin response in *Arabidopsis thaliana* . Genetics 146 (3), 1087–1099. doi: 10.1038/s41598-017-10823-y 9215910 PMC1208037

[B41] Singh V. K. Mangalam A. K. Dwivedi S. Naik S. (1998). Primer premier: program for design of degenerate primers from a protein sequence. Biotechniques 24, 318–319. doi: 10.2144/98242pf02 9494736

[B42] Takagi H. Abe A. Yoshida K. Kosugi S. Natsume S. Mitsuoka C.. (2013). QTL-seq: Rapid mapping of quantitative trait loci in rice by whole genome resequencing of DNA from two bulked populations. Plant J. 74, 174–183. doi: 10.1111/tpj.12105 23289725

[B43] Teichmann T. Muhr M. (2015). Shaping plant architecture. Front. Plant Sci. 6. doi: 10.3389/fpls.2015.00233 PMC4390985 25914710

[B44] Wang X. W. Kole C. (2015). The Brassica rapa Genome: Economic/Academic Importance of Brassica rapa (New York: Springer-Verlag Berlin Heidelberg), 1–2. doi: 10.1007/978-3-662-47901-8

[B45] Wang Y. Li J. Y. (2011). Branching in rice. Curr. Opin. Plant Biol. 14, 94–99. doi: 10.1016/j.pbi.2010.11.002 21144796

[B46] Wang N. Liu Z. Y. Zhang Y. Li C. Y. Feng H. (2018b). Identification and fine mapping of a stay-green gene (*Brnye1*) in pakchoi (*Brassica campestris* L. ssp. *chinensis*). Theor. Appl. Genet. 131, 673–684. doi: 10.1007/s00122-017-3028-8 29209732

[B47] Wang B. Smith S. M. Li J. (2018a). Genetic regulation of shoot architecture. Annu. Rev. Plant Biol. 69, 437–468. doi: 10.1146/annurev-arplant-042817-040422 29553800

[B48] Wang C. Tang S. Zhan Q. Hou Q. Zhao Y. Zhao Q.. (2019). Dissecting a heterotic gene through GradedPool-seq mapping informs a rice-improvement strategy. Nat. Communication 10 (1), 2982. doi: 10.1038/s41467-019-11017-y PMC6611799 31278256

[B49] Wei C. H. Zhu C. Y. Yang L. P. Zhao W. Ma R. X. Li H.. (2019). A point mutation resulting in a 13 bp deletion in the coding sequence of *Cldf* leads to a GA-deficient dwarf phenotype in watermelon. Horticulture Res. 6, 132. doi: 10.1038/s41438-019-0213-8 PMC6885051 31814985

[B50] Wuddineh W. Mazarei M. Zhang J. Y. Poovaiah C. Mann D. Ziebell A.. (2014). Identification and overexpression of *gibberellin 2-oxidase* (*GA2ox*) in switchgrass (*Panicum virgatum* l.) for improved plant architecture and reduced biomass recalcitrance. Plant Biotechnol. J. 13, 636–647. doi: 10.1111/pbi.12287 25400275

[B51] Xia T. Li Na. Dumenil J. Li J. Kamenski A. Bevan M. W.. (2013). The ubiquitin receptor DA1 interacts with the E3 ubiquitin ligase DA2 to regulate seed and organ size in arabidopsis. Plant Cell 25, 3347–3359. doi: 10.1105/tpc.113.115063 24045020 PMC3809536

[B52] Xu X. Ji J. Xu Q. Qi X. Weng Y. Chen X. (2018). The major-effect quantitative trait locus *CsARN6.1* encodes an AAA ATPase domain-containing protein that is associated with waterlogging stress tolerance by promoting adventitious root formation. Plant J. 93, 917–930. doi: 10.1111/tpj.13819 29315927

[B53] Xu J. Zha M. Li Y. Ding Y. Chen L. Ding C.. (2015). The interaction between nitrogen availability and auxin, cytokinin, and strigolactone in the control of shoot branching in rice (*Oryza sativa* l.). Plant Cell Rep. 34, 1647–1662. doi: 10.1007/s00299-015-1815-8 26024762

[B54] Yang S. J. Tian X. X. Wang Z. Y. Wei X. C. Zhao Y. Y. Su H. N.. (2021). Fine mapping and candidate gene identification of a white flower gene *BrWF3* in Chinese cabbage (*Brassica rapa* l. ssp. *pekinensis*). Front. Plant Sci. 12. doi: 10.3389/fpls.2021.646222 PMC8138439 34025693

[B55] Yang H. Xue Q. Zhang Z. Z. Du J. Y. Yu D. Y. Huang F. (2018). GmMYB181, a soybean R2R3-MYB protein, increases branch number in transgenic *Arabidopsis* . Front. Plant Sci. 9. doi: 10.3389/fpls.2018.01027 PMC6056663 30065741

[B56] Zhang Q. Deng A. W. Xiang M. (2022). The root hair development of pectin polygalacturonase PGX2 activation tagging line in response to phosphate deficiency. Front. Plant Sci. 13. doi: 10.3389/fpls.2022.862171 PMC9108675 35586221

[B57] Zhou B. Peng D. Lin J. Huang X. Peng W. He R.. (2011). Heterologous expression of a gibberellin 2-oxidase gene from *Arabidopsis thaliana* enhanced the photo-synthesis capacity in *Brassica napus* l. J. Plant Biol. 54, 23–32. doi: 10.1007/s12374-010-9139-2

[B58] Zou C. Wang P. Xu Y. (2016). Bulked sample analysis in genetics, genomics and crop improvement. Plant Biotechnol. J. 14, 1941–1955. doi: 10.1111/pbi.12559 26990124 PMC5043468

