# Supplementary figures and images for: Identification of two tandem genes associated with primary rosette branching in flowering Chinese cabbage
Source: Front Plant Sci. 2022 Dec 19;13:1083528. doi: 10.3389/fpls.2022.1083528 (PMC9806259; doi:10.3389/fpls.2022.1083528)

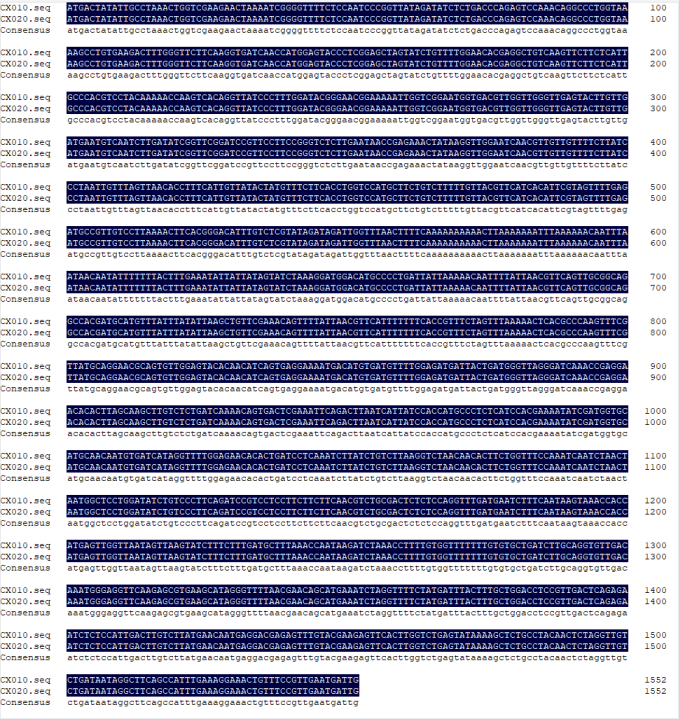

Supplement: Supplementary Figure 1 — The full-length of gene BraA07g041560.3C. [file Image_1.tif]

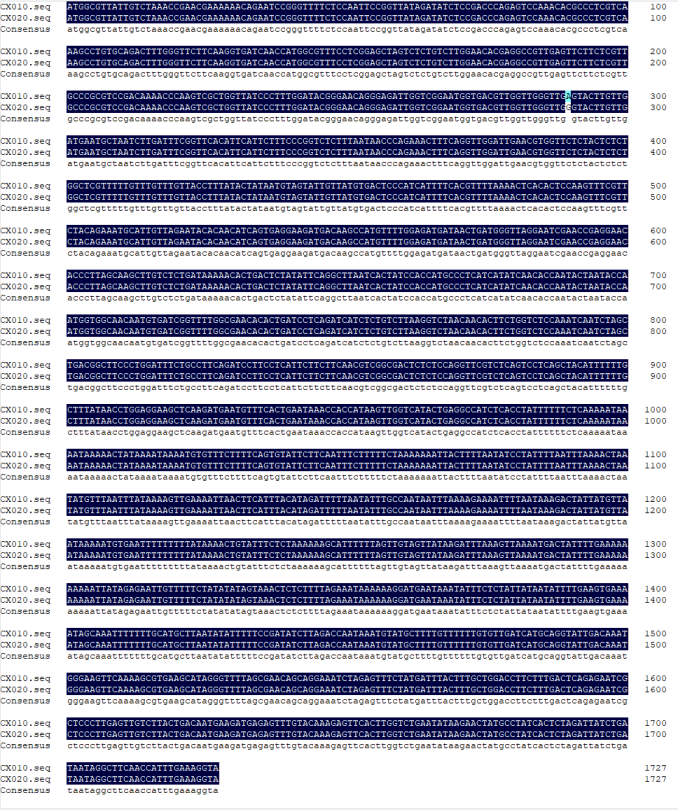

Supplement: Supplementary Figure 2 — The full-length of gene BraA07g041570.3C. [file Image_2.tif]

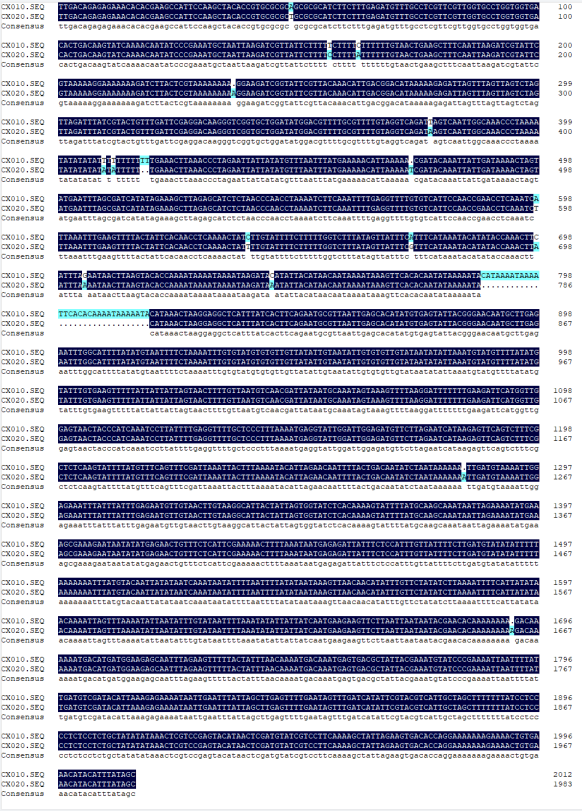

Supplement: Supplementary Figure 3 — The promoter sequence of BraA07g041560.3C. [file Image_3.tif]

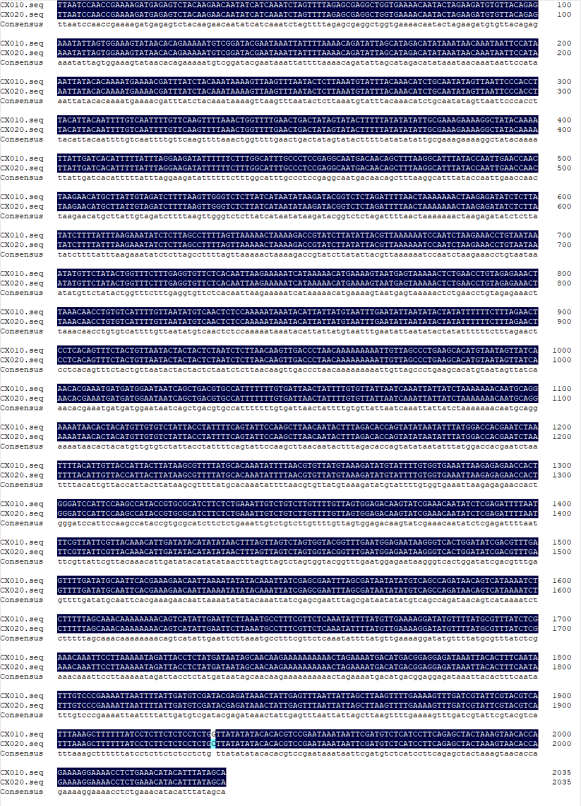

Supplement: Supplementary Figure 4 — The promoter sequence of BraA07g041570.3C. [file Image_4.tif]
